# Pressure-Free Assembling of Poly(methyl methacrylate) Microdevices via Microwave-Assisted Solvent Bonding and Its Biomedical Applications

**DOI:** 10.3390/bios11120526

**Published:** 2021-12-20

**Authors:** Kieu The Loan Trinh, Woo Ri Chae, Nae Yoon Lee

**Affiliations:** 1 Department of Industrial Environmental Engineering, Gachon University, 1342 Seongnam-daero, Sujeong-gu, Seongnam-si 13120, Korea; trinhloan@gachon.ac.kr; 2 Department of BioNano Technology, Gachon University, 1342 Seongnam-daero, Sujeong-gu, Seongnam-si 13120, Korea; olympia@gachon.ac.kr

**Keywords:** poly(methyl methacrylate), acetic acid, solvent bonding, microwave-assisted bonding, PMMA microdevices

## Abstract

Poly(methyl methacrylate) (PMMA) has become an appealing material for manufacturing microfluidic chips, particularly for biomedical applications, because of its transparency and biocompatibility, making the development of an appropriate bonding strategy critical. In our research, we used acetic acid as a solvent to create a pressure-free assembly of PMMA microdevices. The acetic acid applied between the PMMA slabs was activated by microwave using a household microwave oven to tightly merge the substrates without external pressure such as clamps. The bonding performance was tested and a superior bond strength of 14.95 ± 0.77 MPa was achieved when 70% acetic acid was used. Over a long period, the assembled PMMA device with microchannels did not show any leakage. PMMA microdevices were also built as a serpentine 2D passive micromixer and cell culture platform to demonstrate their applicability. The results demonstrated that the bonding scheme allows for the easy assembly of PMMAs with a low risk of clogging and is highly biocompatible. This method provides for a simple but robust assembly of PMMA microdevices in a short time without requiring expensive instruments.

## 1. Introduction

The advancement of microfabrication techniques has resulted in the transition from batch analysis in laboratories to portable, cost-effective, and highly efficient miniaturized microfluidic systems. Several research areas have taken advantage of microfluidic systems such as microreactors, biosensors, or organ-on-chip [1,2,3].

The material and the sealing method chosen are critical for the reliable performance of microfluidic chips. Silicon-based materials, such as silicon wafers, glass, or polydimethylsiloxane (PDMS), have been widely used because they can be directly applied following the microfabrication steps of photolithography or soft lithography via oxygen plasma bonding, which endows their surfaces with a high oxygen content to form a strong Si-O-Si linkage or anodic bonding [4,5,6]. However, glass and silicon wafers are susceptible to breakage and the gas-permeable nature of PDMS limits its application in high temperatures due to the risk of reagent evaporation [7].

To compensate for the limitations associated with the use of silicon-based materials, thermoplastics such as poly(methyl methacrylate) (PMMA), polycarbonate (PC), and polyethylene terephthalate (PET) have been widely used. Because of its low cost, good optical clarity, biocompatibility, and durability, PMMA, in particular, has gained popularity, and numerous bonding strategies have been developed to construct PMMA-based microfluidic devices. PMMAs can be assembled through thermal bonding by increasing the bonding temperature above the glass transition temperature to soften the chains and interlock the two surfaces [8,9]. This is an intuitively simple method that does not require any additional adhesive reagents, but it is typically only applicable between homogeneous plastics or plastics with similar glass transition temperatures, as the high heat and pressure can cause deformation of the microfluidic channels. Chemically modifying the plastic surfaces can lower the bonding temperature hence reducing the chance of channel deformation. However, the modified surfaces may not be suitable for the microdevices’ intended applications, and it frequently results in a chemically non-homogeneous channel surface [10,11,12].

Solvent-assisted bonding is performed by dissolving the polymer chains at the bonding interface to tightly interlock the plastic surfaces without requiring harsh temperatures, and the solvents can be easily evaporated to leave the original plastic surface [13,14,15,16]. Appropriate solvents can aid in the assembly of PMMA microdevices, but the solvent composition and bonding conditions must be tightly controlled to avoid deformation or clogging of the microchannels. Attempts were made to limit the solvents’ negative effect on the integrity of the microchannels; Ling et al. have constructed a clog-free PMMA microdevice by selectively coating a hydrophobic material on the channel surface before the ethanol-assisted solvent bonding [17]. The hydrophobic coating successfully prevented ethanol from damaging the channel wall, but the surface became hydrophobic and lost its original properties as a result. In previous studies, we replaced commonly used solvents such as acetone, ethanol, or an ethanol/isopropyl alcohol mixture with a dilute acetic acid solution, which resulted in clog-free and deformation-free assembly of PMMA microdevices without the need for any other protective coating material. By using the diluted acetic acid solution, PMMA microdevices were successfully bonded within 20 min at room temperature, or merely 30 s under UV treatment [18,19].

Furthermore, microwave-assisted thermoplastic bonding, particularly PMMA, has been reported for effective microdevice assembly. Although PMMA does not absorb microwaves and the bulk temperature cannot be raised sufficiently to fuse the substrates, some microwave-sensitive materials can be incorporated to support the melting of PMMA via radiation-induced heating [20,21,22,23]. In some studies, thin layer deposition of metal layers, particularly gold, was used to generate localized heating under the microwave [22,24]. The PMMA substrates were successfully fused by forming tight bonding between either the patterned metal intermediate layer and PMMA substrates or PMMA and PMMA. However, because this method involves the deposition of expensive metals, it is rather expensive and overly complicated for producing disposable microdevices. Rahbar et al. successfully formed tight sealing of PMMA devices by using poor solvents between the PMMA substrates and clamping them tightly using metal binders [23]. These metal clamps are made of a ferromagnetic material that, when microwaved, emits enough heat to easily melt and fuse the PMMAs with the help of the solvents. Although more advantageous from an economic point of view given that the metal clamps are reusable, overheating of the clamps should be carefully watched, and because effective heating is only generated around the clamped regions, it is expected that bonding a large area with complicated channel structures will be difficult.

In this study, we were able to achieve uniform bonding and high bond-strength without using any external pressure by performing acetic acid-assisted assembly of PMMA microdevices under microwave irradiation. The peripheral groove at the microdevice’s boundary was engraved to efficiently retain acetic acid during microwave irradiation and thus prevent evaporation, resulting in uniform bonding across the entire surface [25]. Acetic acid-assisted bonding was exploited in our previous studies both in room temperature condition or under UV irradiation for the fabrication of the PMMA microdevices with reliable bonding performance, but the PMMAs were pressurized during the bonding process [18,19]. Surprisingly, the use of a common household microwave and peripheral grooves in this study eliminated the need for clamps or other microwave absorbers like polymers or metals for the stable bonding of PMMAs. After the bonding process, the bond strength measurement and leak test were evaluated to confirm the successful bonding with the clog-free joining of the PMMA device. As a proof of concept, the fabricated PMMA microdevices were used in micro-mixing and human cell culture, demonstrating their bond quality and applicability.

## 2. Materials and Methods

### 2.1. Materials

Goodfellow provided the PMMA substrates (2 mm in thickness) (Coraopolis, PA, USA). Sigma-Aldrich provided the acetic acid (99.7%) (St. Louis, MO, USA). All bonding experiments and analytical measurements were performed with acetic acid solutions diluted in deionized (DI) water to the appropriate concentrations. Mesenchymal stem cells (MSCs), human umbilical vein endothelial cells (HUVECs), mesenchymal stem cell medium, and endothelial cell medium were acquired from Sciencell (Carlsbad, CA, USA). Thermo Fisher Scientific provided calcein-AM and ethidium homodimer-1 (EthD-1) for the cell viability/cytotoxicity test (Waltham, MA, USA). Dow Corning supplied a silicone tube (i.d. 1.0 mm, o.d. 2.0 mm) (Midland, MI, USA). A commercial microwave oven (Samsung RE-C23XM) was used.

### 2.2. Bonding Mechanism

**Figure 1:**
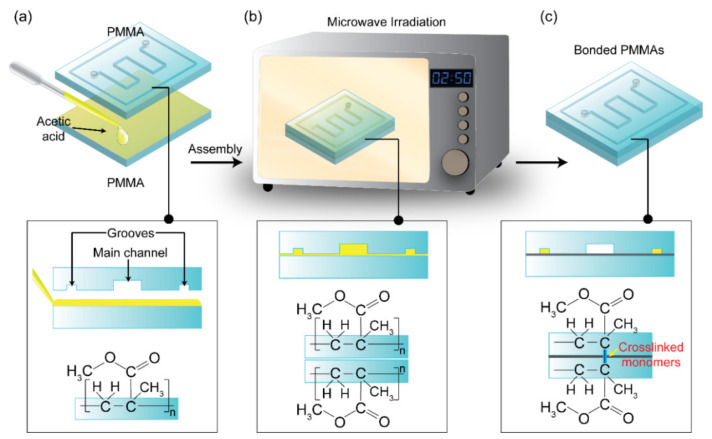
Schematic of a microwave-assisted solvent bonding using acetic acid for assembling a PMMA microdevice. (**a**) Acetic acid was applied on one PMMA substrate and then carefully pressed together. (**b**) Two PMMA substrates with acetic acid were placed in a microwave oven for 2 min 50 s without the need for any external pressure for permanent bonding. (**c**) PMMAs were bonded to form a microfluidic chip after the bonding process.

Figure 1 shows the overall scheme for bonding two PMMA substrates using an acetic acid treatment followed by microwave irradiation. The main microchannel and the peripheral groove are engraved on PMMA. First, approximately 15 μL of the acetic acid solution was applied to the PMMA substrate (4 cm × 4 cm), and another PMMA substrate was placed on top, as shown in Figure 1a. It should be noted that when the acetic acid solution comes into contact with the surface, it must completely cover it while avoiding the formation of air bubbles. Then the acetic acid-treated PMMA substrates were placed on the turntable of the household microwave oven (700 watts, 2450 MHz) and irradiated with microwave for 2 min 50 s to facilitate bonding (Figure 1b). The use of acetic acid as a solvent helped to loosen the PMMA polymers at the bonding interface, and the heat from the microwave aided in the bonding of the two PMMAs via cross-linked monomers of PMMA polymer chains. Finally, as shown in Figure 1c, the PMMAs formed a permanent bond and were removed from the microwave oven. Using the air gun through the inlets and outlets, the remaining acetic acid solution can be easily removed from the microchannels. Additionally, the hypothesized chemical reaction expected to occur between the PMMA substrates when treated with acetic acid and microwave irradiation is shown in Figure 1.

### 2.3. Contact Angle Measurement

The surface wettability of the acetic acid-treated PMMAs following the microwave irradiation was evaluated by water contact angle measurements. For the measurements, PMMA substrates (40 mm × 20 mm) were covered with 10, 20, 30, 40, 50, 60, 70, and 100% acetic acid solution and immediately microwave-treated for 2 min 50 s. It is worth noting that the original concentration of acetic acid was 99.7%, and this concentration was considered as 100% for the convenience of the calculation. After the microwave treatment, the PMMA substrates were carefully dried with compressed air before the water contact angles were measured. A Phoenix 300 contact angle analyzer (Surface Electro Optics, Suwon-si, Gyeonggi-do, Korea) was used to measure water contact angle at the Core Facility for Bionano Materials at Gachon University. The machine was used to measure the contact angle by depositing static water droplets onto the substrates. The Image-Pro 300 software was used to analyze the results. For further evaluation, five measurements were taken and averaged. For comparison, the water contact angle was also measured on the surface of a pristine PMMA and microwave-treated pristine PMMA as references.

### 2.4. Bond Strength Analyses

To evaluate the bonding performance, the bond strength was analyzed using a texture analyzer (QST 25, Brookfield, Middleborough, MA, USA). In brief, two PMMA substrates (40 mm × 20 mm × 2 mm) were bonded at a partially overlapped region with an acetic acid solution (50, 60, and 70%) and microwave-treated for 2 min 50 s. To measure bond strength, partially bonded PMMAs with through-holes were prepared with a drilling machine, and twines were inserted and tightly fastened to pull the substrates in the opposite direction. The partially bonded PMMA substrates were fixed to the analyzer and pulled at a speed of 100 mm min^−1^ for measurement. The overlapped lengths of the two PMMAs ranged between 0.75 and 2 mm. All the experiments were triplicated to confirm the bonding reproducibility.

### 2.5. Bonded PMMA Microdevice as a Passive Micromixer

To confirm the feasibility of exploiting the introduced bonding method to assemble a functioning microdevice, a simple serpentine passive micromixer was fabricated. Using a computer numerical control (CNC) machine, a serpentine microchannel (500 μm in depth, 1000 μm in width, and 20 cm total length of mixing area) was engraved on the PMMA substrate. The ink samples were introduced into the micromixer at a rate of 1 mL min^−1^ using a double port syringe pump (KDS 200, KD Scientific, Hayward, CA, USA). The red and green ink solutions were diluted in DI water and used to evaluate the performance of the micromixer. A smartphone camera (a native Camera app, 12MP, 1× or 26 mm-equivalent, main camera at f/1.6 at Auto ISO 250, size 3841 × 2881, shutterspeed 1/60 s) was used to acquire the optical images.

### 2.6. Bonded PMMA Microdevice as a Platform for Human Cell Culture

Using the scheme presented in this study, a PMMA microdevice with straight channels was created by bonding a flat PMMA substrate with an engraved PMMA substrate. The exterior of the assembled microdevice was wiped with 70% ethanol and sterilized for 1 h on a clean bench under UV light. MSCs and HUVECs were separately cultured in T-75 flasks at 37 °C and 5% CO_2_ level while keeping the medium fresh by exchanging them every 1–2 days. The cells were trypsinized for detachment and then centrifuged before being introduced into the PMMA microchannels after being diluted in a fresh medium. The medium was exchanged every day until the viability/cytotoxicity test using calcein-AM and EthD-1.

## 3. Results and Discussion

### 3.1. Contact Angle Measurement

**Figure 2:**
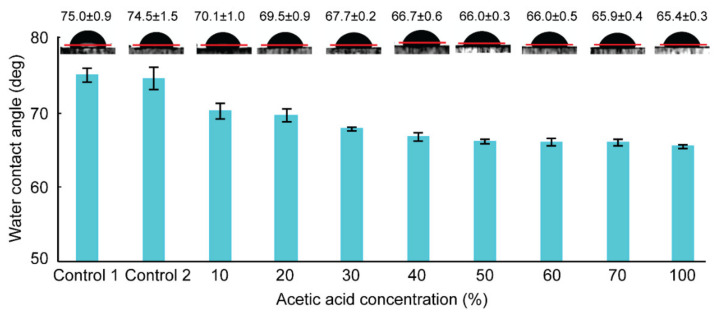
Water contact angles measured on flat PMMA surfaces treated with different concentrations of acetic acid followed by microwave irradiation. Each measurement was repeated five times.

Figure 2 depicts the change in surface wettability after acetic acid and microwave treatment. All of the PMMA substrates were treated with acetic acid solutions in varying concentrations before being microwave irradiated for 2 min 50 s. As shown in Figure 2, the water contact angles of the microwave-treated PMMA (Control 2) was approximately 74.5 ± 1.5°, which closely matched the values from the pristine PMMA (75.0 ± 1.0°) (Control 1). Because of PMMA’s microwave transparency, the microwave treatment alone did not affect the PMMA surface. However, when acetic acid was added, the contact angle values began to decrease from 70.1 ± 1.0° to 65.4 ± 0.3° as the concentrations of acetic acid solution were increased from 10 to 100%, respectively. Based on the results, we can assume that the acetic acid molecules dissolved and penetrated the PMMA chains, increasing the surface’s wettability with water. The similarity of the Hildebrand solubility parameters of acetic acid and PMMA, as mentioned in our previous study [18], can explain this. From the results, we can predict that a heavier dissolution of PMMA chains to the solvent occurs at higher acetic acid concentrations and the acetic acids penetrated the deeper layer of the PMMA substrates. However, at lower acetic acid concentrations, the dissolution of PMMA to the solvent decreases, as indicated by the smaller change in contact angles. As a result, by adjusting the solvent concentrations, a suitable solvent condition that can prevent clogging or deformation while maintaining a strong bonding can be found.

### 3.2. Bonding Performance: Effect of Acetic Acid Concentrations and Addition of Grooves

**Figure 3:**
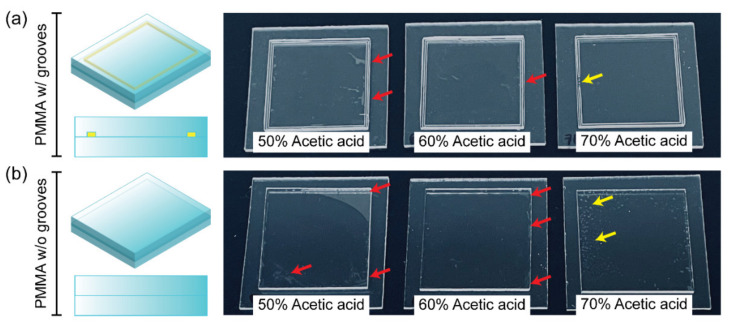
(**a**) Results of PMMA-PMMA bonding performed under various concentrations of acetic acid solutions (50%, 60%, and 70%) in the presence of the peripheral grooves. (**b**) Results of PMMA-PMMA bonding performed under various concentrations of acetic acid solutions (50%, 60%, and 70%) without peripheral groove. Red arrows indicate the unbonded areas, and yellow arrows indicate the melted areas.

Figure 3 shows the bonding performance of two PMMA substrates using different concentrations of acetic acid (50–70%). In this experiment, two types of PMMA substrates with or without peripheral grooves were used for bonding, and the groove dimension was approximately 1000 μm × 500 μm (width × depth), placed 1 mm from the substrate’s edges. All experiments were carried out under microwave irradiation for 2 min and 50 s. As a result, the bonded area has noticeably increased with the increasing acetic acid concentration in both cases of PMMA substrates with or without peripheral grooves. PMMA substrates with grooves obtained more uniform bonding even near the edges, as shown in Figure 3a, than those without grooves (Figure 3b). In terms of channel integrity and bond coverage, the 60% acetic acid achieved the highest bonding quality. The previous study describes the addition of grooves and their benefits during the solvent bonding process in detail [25]. The groove at the periphery of the PMMA substrate has behaved as a reservoir to retain solvent and prevent solvent evaporation during microwave irradiation and heating. In the case of bonding the PMMA substrates without grooves using 50 and 60% acetic acid, more unbonded areas were created especially near the edges due to solvent evaporation. For 70% acetic acid, the combination of a faster dissolution rate at higher concentrations and microwave heating melted the substrates excessively. According to the findings, 60% acetic acid was found to be the optimum condition for achieving the successful and homogeneous bonding of PMMAs with 2 min and 50 s of microwave irradiation.

### 3.3. Bond Strength Measurement

**Figure 4:**
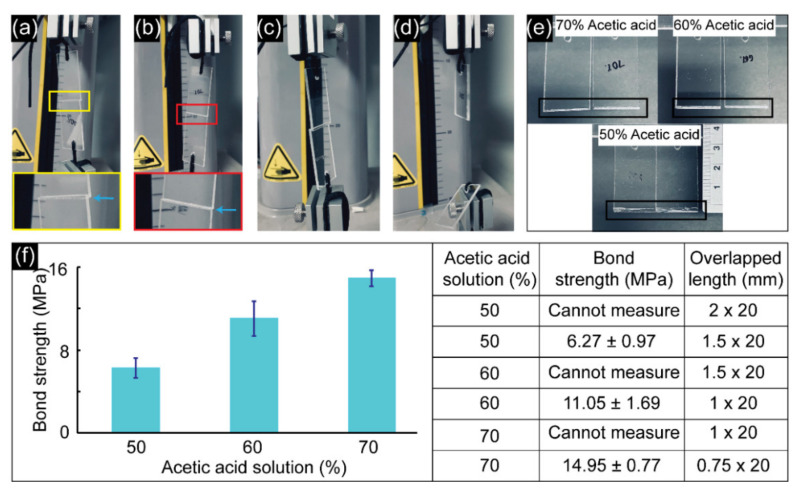
Results of bond strength measurement. (**a**,**b**) PMMA assemblies with the overlapped length of 1 mm and 0.75 mm, respectively, using 70% acetic acid before the detachment. The yellow box shows the enlarged picture of the bonded PMMAs with the overlapped length of 1 mm, and the red box with the overlapped length of 0.75 mm. (**c**,**d**) PMMA assemblies with the overlapped length of 1.5 mm using 50% acetic acid before and after the detachment, respectively. (**e**) Enlarged photos showing the bonded area after the detachment of PMMA assemblies using different acetic acid concentrations (50%, 60%, and 70%). (**f**) Results of bond strength were measured at different acetic acid concentrations (50%, 60%, and 70%). All experiments were performed in triplicate.

**Figure 5:**
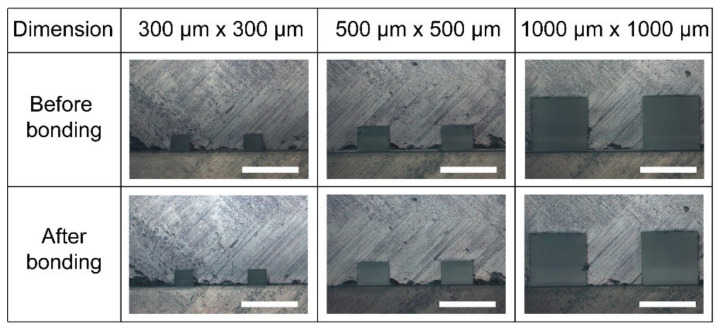
Bright-field photographs of the cross-sectional views of square 300 × 300, 500 × 500, and 1000 × 1000 µm^2^ microchannels engraved on the PMMA substrates before and after the bonding. 60% acetic acid solution was used to achieve the bonding. Scale bars: 1000 μm.

Figure 4 shows the results of bond strength measurements of the PMMA substrates (20 mm × 40 mm × 2 mm). Figure 4a,b shows pictures of the bonded PMMAs using 70% acetic acid in which the overlapped lengths were 1 and 0.75 mm, respectively. Figure 4c,d depicts images of the bonded PMMAs treated with 50% acetic acid before and after detachment, with an overlapped length of 1.5 mm. Figure 5e depicts the PMMAs that were bonded using various acetic acid concentrations after detachment (50–70%). From these results, when the acetic acid concentration was increased from 50 to 70% for bonding PMMAs, the overlapped lengths must be scaled down from 1.5 to 0.75 mm to be able to detach the bonded PMMAs using the analyzer. In other words, bond strengths for PMMAs bonded with 50–70% acetic acid with an overlapped length of more than 1.5 mm could not be measured. When bonding with acetic acid and a microwave, the overlapped length had to be much smaller than in previous studies [18,19], otherwise the bonded PMMAs failed to detach. Figure 4f shows the bond strength after bonding using 50–70% acetic acid. Briefly, the bond strengths increased with the increasing acetic acid concentration, which was indicated by the measured values of 6.26 ± 0.97, 11.05 ± 1.69, and 14.95 ± 0.77 MPa for 50, 60, and 70% acetic acid, respectively. The highest bond strength of 14.95 ± 0.77 MPa obtained using 70% acetic acid was greater than that obtained in previous studies for bonding PMMA microdevices [18,19]. However, to avoid clogging of the microchannels, 60% acetic acid was chosen as the best bonding condition for future experiments.

### 3.4. Microchannel Profile: Cross-Sectional View of the Microchannel

Figure 5 shows the optical images of the cross-section of the microchannels created by bonding PMMA substrates using 60% acetic acid and microwave irradiation for 2 min 50 s. Using CNC milling, various dimensions of square microchannels were engraved on PMMA substrates using different end mill sizes. The results show that the microchannel geometries were nearly identical before and after bonding with the proposed method. Furthermore, the clog-free bonding of the microchannel was confirmed at a size as small as 300 μm × 300 μm. As a weak solvent, acetic acid did not strongly melt or damage the PMMA surface even under the influence of microwave irradiation when compared with other strong solvents. Furthermore, as stated in the bonding procedure, no external pressure was used during microwave irradiation, which may have contributed to the preservation of the original microchannel geometry during the bonding process. As a result, the introduced method can also be used to assemble a PMMA microdevice with small microstructures.

### 3.5. Leak Test

**Figure 6:**
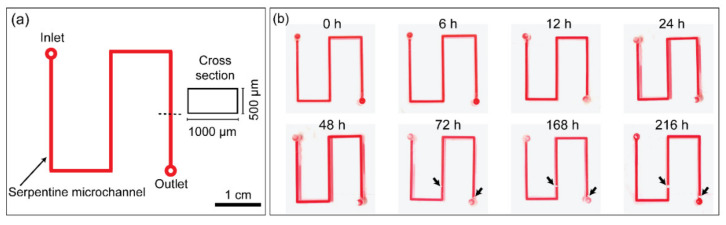
(**a**) Illustration of the microdevice used for the leak test. (**b**) Results of the leak test performed when observed over a period from 0 to 216 h. The black arrows indicate ink that was lost due to evaporation.

Figure 6 shows the results of the leak test performed to confirm the stability of the PMMA microdevice fabricated using the introduced bonding method. Figure 6a shows the design of the microdevice (4 cm × 4 cm) with a simple serpentine microchannel (1000 μm × 500 μm), which was sealed for performing the leak test. Following the bonding process, the ink sample was introduced and observed inside the microdevice for 216 h (up to 9 days) at room temperature (Figure 6b). Despite the adhesive tapes over the inlet and outlet for enclosing the microchannel, the ink began to evaporate 72 h after injection. However, as demonstrated by the results, the ink was stably maintained and held inside the microchannel with no leakage into the bonded interface. Moreover, using the introduced bonding method, the multilayered microdevice was also successfully fabricated as shown in Figure S1. Briefly, three layers of PMMA including two 2 mm thick PMMAs and one 0.15 mm thick PMMA were successfully bonded using the introduced method in a single operation. These results demonstrated that the PMMA bonding method suggested in this study can be adopted to create microdevices with tight bonding for long-term use.

### 3.6. Bonded PMMA Microdevice Used as a Planar Passive Micromixer

**Figure 7:**
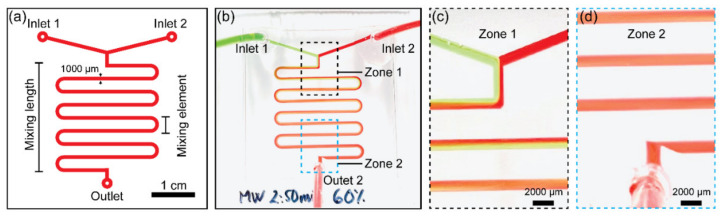
(**a**) A detailed illustration of the planar serpentine passive micromixer used for mixing the two ink solutions. (**b**) Optical image of a planar serpentine passive micromixer in operation. (**c**) Enlarged photo of “Zone 1” marked in (**b**) showing the mixing pattern near the inlet at a flow rate of 1 mL min^−1^. (**d**) Enlarged photo of “Zone 2” marked in (**b**) showing the mixing pattern near the outlet at a flow rate of 1 mL min^−1^.

Micromixers are one of the critical components within microfluidic biomedical systems, micro-total-analysis systems (μTAS), and lab-on-a-chip devices. A simple serpentine micromixer was built to demonstrate the applicability of the introduced bonding method, as shown in Figure 7. The microchannel had a dimension of 500 μm × 1000 μm, and the device was built on 2 mm thick PMMA substrates. Two syringe pumps were used to introduce the diluted green- and red-color inks into the bonded micromixer. Figure 7a illustrates the design of the microdevice including a serpentine microchannel as a passive mixer. For both syringe pumps, the flow rate was set to 1 mL min^−1^. As a result, as shown in Figure 7b, two liquid streams were successfully mixed using the bonded micromixer. Furthermore, the bonded mixer withstood the pressure from the syringe pump without leaking or logging from the microdevice during the mixing process. As expected, near the inlets, two liquids moved along the microchannel following laminar flow (Figure 7c), but they were considerably well-mixed at the outlet zone after going through the mixing area (Figure 7d). These findings suggest that the acetic acid- and microwave-treated microdevices can be used to fabricate micromixers with high potential for use in a variety of biomedical systems-related studies.

### 3.7. Bonded PMMA Microdevice for Human Cell Culture

**Figure 8:**
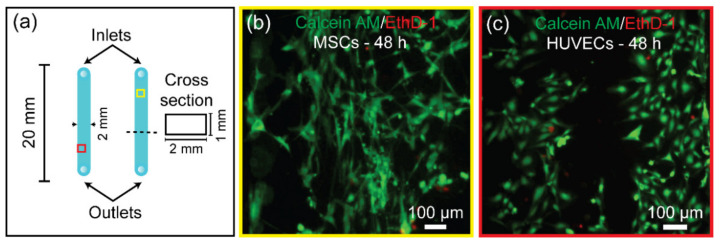
(**a**) A detailed design of the channel dimension used in cell culture. Fluorescent microscopic images of calcein-AM/EthD-1 stained (**b**) MSCs and (**c**) HUVECs to evaluate cell viability. The live/dead cells are indicated by calcein-AM for live cells (green) and EthD-1 for dead cells (red).

PMMA possesses good biocompatibility and has been widely used for biomedical applications such as bone cement, disposable hemocytometers, organ-on-chips, or microfluidic chips for evaluation of a drug performance [26,27,28]. As a result, any negative effect of the microwave-assisted acetic acid bonding strategy on cell viability must be investigated to validate its biocompatibility. Before performing the cell experiment, the PMMA surface was characterized using the Fourier transform infrared spectroscopy (FTIR). The results confirmed that the acetic acid treatment followed by microwave treatment did not change the chemical properties of PMMA (Figure S2). Figure 8 depicts the results of cultured MSCs and HUVECs on a PMMA device with straight channels bonded using the method described in this study. Figure 8a depicts the design of the cell culture channels, which were created by engraving the PMMA substrate with a 2 mm drill to cut 1 mm in depth. The viability of the cells was tested by staining the cells on the channels using calcein-AM for live cells and EthD-1 for dead cells (Figure 8b,c). The fluorescence imaging of the stained cells demonstrated that the cells grown in the channels for 48 h were mostly alive, which was indicated by the calcein-AM stained cells (green). It should be noted that before inserting cells into the PMMA device, it is recommended that any acetic acid remnants in the channel be evaporated by leaving the device on a hotplate set to 80 °C for a couple of hours. We confirm that the resulting surface is highly biocompatible after properly removing the acetic acid in the PMMA microdevice, and that this bonding strategy can provide a good alternative to cheaply fabricate PMMA microdevices for cell-related studies using a simple household instrument.

## 4. Conclusions

We presented a simple and robust strategy for assembling PMMA-based microdevices using acetic acid as a solvent, aided by the use of a microwave in this study. The method is simple, quick, and does not require external pressure to tightly hold the device during the bonding process. It is also simple to implement, requiring only a common household instrument. Our current study suggests that when microwave and peripheral grooves are adopted together, a uniform and strong bonding is possible even without the presence of pressure. The bond strength achieved was as high as 14.95 ± 0.77 MPa when using 70% acetic acid, which is the highest among our previous acetic acid-based PMMA bonding strategies. This fast and strong PMMA bonding method is a promising option for producing PMMA microdevices in low-resource settings. We also validated its applicability by building a serpentine micromixer and performing a biocompatibility test. The resulting PMMA microdevices exhibited no leakage over a long period and were beneficial to cell survival.

## Data Availability

The data presented in this study are available on request from the corresponding author.

## Supplementary Materials

The following are available online at https://www.mdpi.com/article/10.3390/bios11120526/s1, Figure S1: Multilayer bonding of a PMMA microdevice by acetic acid and microwave treatment. (a) Illustration of the device design where PMMA layers 1-3 are bonded to form top microchambers and bottom microchannels separated by a thin PMMA film in the middle. (b) Pictures of the fabricated multilayered PMMA microdevice with inks loaded; Figure S2: Comparative FTIR spectra of bare PMMA, microwave-treated PMMA, and acetic acid/microwave-treated PMMA.
