# Supplementary material for: Pressure-Free Assembling of Poly(methyl methacrylate) Microdevices via Microwave-Assisted Solvent Bonding and Its Biomedical Applications
Source: Biosensors (Basel). 2021 Dec 20;11(12):526. doi: 10.3390/bios11120526 (PMC8699324; doi:10.3390/bios11120526)
Supplement: Supplementary file 1 [file biosensors-11-00526-s001.zip › biosensors-1488607-supplementary.pdf]

biosensors

MDPI

Supplementary Materials

Pressure-free assembling of poly(methyl methacrylate) microdevices via microwave-assisted solvent bonding and its biomedical applications

Kieu The Loan Trinh [1], Woo Ri Chae [2] and Nae Yoon Lee [2,*]

[1] Department of Industrial Environmental Engineering, Gachon University, 1342 Seongnam-daero, Sujeong-gu, Seongnam-si, Gyeonggi-do, 13120, Korea; tktloan@gmail.com (K.T.L.T.)
[2] Department of BioNano Technology, Gachon University, 1342 Seongnam-daero, Sujeong-gu, Seongnam-si, Gyeonggi-do, 13120, Korea.; findingwoori@gmail.com (W.R.C.); nylee@gachon.ac.kr (N.Y.L)
* Correspondence: nylee@gachon.ac.kr

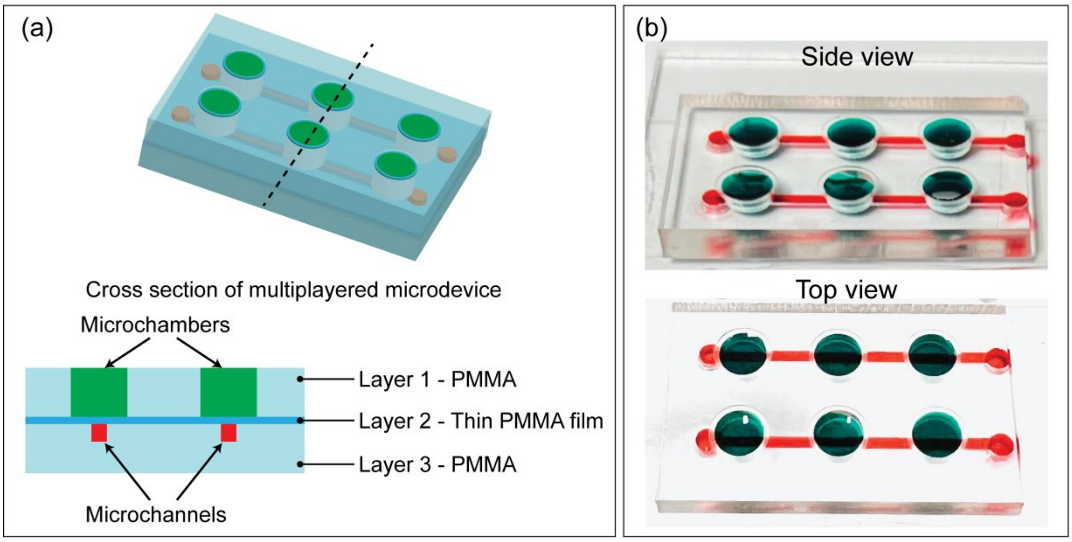

Figure S1. Multilayer bonding of a PMMA microdevice by acetic acid and microwave treatment. (**a**) Illustration of the device design where PMMA layers 1-3 are bonded to form top microchambers and bottom microchannels separated by a thin PMMA film in the middle. (**b**) Pictures of the fabricated multilayered PMMA microdevice with inks loaded.

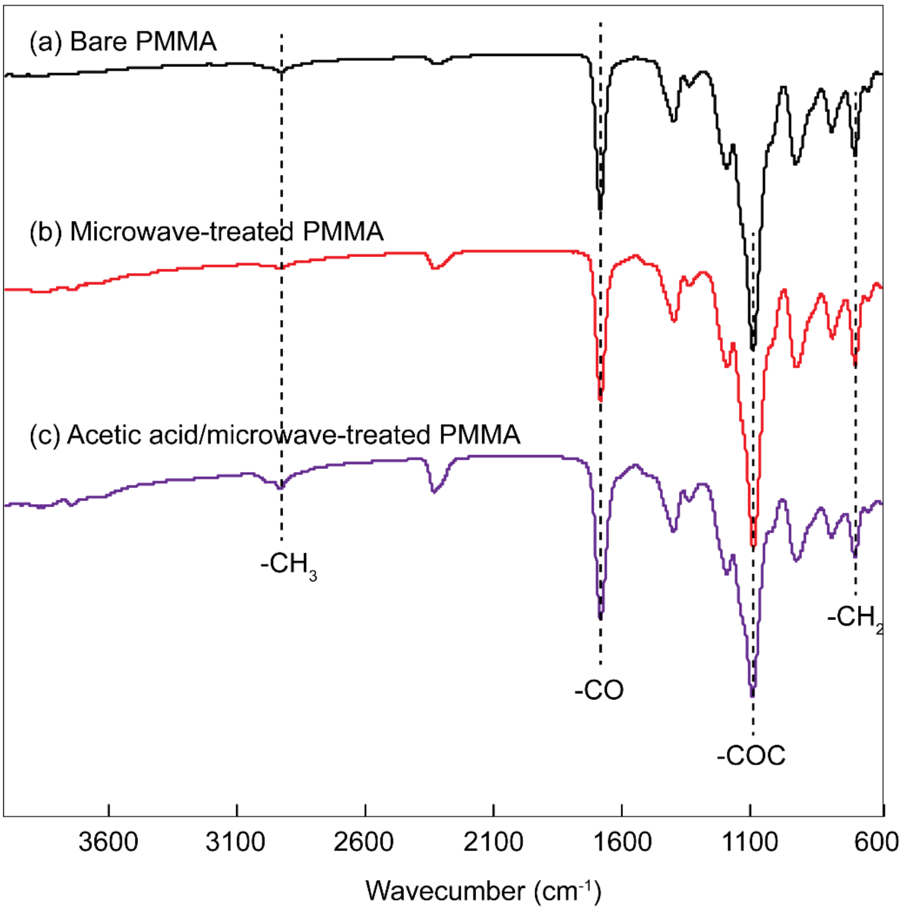

Figure S2. Comparative FTIR spectra of bare PMMA, microwave-treated PMMA, and acetic acid/microwave-treated PMMA.
